# Imaging Challenges in Patients with Severe Aortic Stenosis and Heart Failure: Did We Find a Way Out of the Labyrinth?

**DOI:** 10.3390/jcm11020317

**Published:** 2022-01-10

**Authors:** Birgid Gonska, Dominik Buckert, Johannes Mörike, Dominik Scharnbeck, Johannes Kersten, Cesare Cuspidi, Wolfang Rottbauer, Marijana Tadic

**Affiliations:** 1 Klinik für Innere Medizin II, Universitätsklinikum Ulm, Albert-Einstein Allee 23, 89081 Ulm, Germany; birgid.gonska@uni-ulm.de (B.G.); dominik.burkert@uni-ulm.de (D.B.); johannes.moerike@uni-ulm.de (J.M.); Dominik.Scharnbeck@uni-ulm.de (D.S.); Johannes.kersten@uni-ulm.de (J.K.); wolfgang.rottbauer@uni-ulm.de (W.R.); 2 Department for Internal Medicine, University of Milan-Bicocca, 20126 Milan, Italy; cesare.cuspidi@unimib.it

**Keywords:** aortic stenosis, echocardiography, cardiac magnetic resonance, computed tomography, TAVR

## Abstract

Aortic stenosis (AS) is the most frequent degenerative valvular disease in developed countries. Its incidence has been constantly rising due to population aging. The diagnosis of AS was considered straightforward for a very long time. High gradients and reduced aortic valve area were considered as “*sine qua non*” in diagnosis of AS until a growing body of evidence showed that patients with low gradients could also have severe AS with the same or even worse outcome. This completely changed the paradigm of AS diagnosis and involved large numbers of parameters that had never been used in the evaluation of AS severity. Low gradient AS patients may present with heart failure (HF) with preserved or reduced left ventricular ejection fraction (LVEF), associated with changes in cardiac output and flow across the aortic valve. These patients with low-flow low-gradient or paradoxical low-flow low-gradient AS are particularly challenging to diagnose, and cardiac output and flow across the aortic valve have become the most relevant parameters in evaluation of AS, besides gradients and aortic valve area. The introduction of other imaging modalities in the diagnosis of AS significantly improved our knowledge about cardiac mechanics, tissue characterization of myocardium, calcium and inflammation burden of the aortic valve, and their impact on severity, progression and prognosis of AS, not only in symptomatic but also in asymptomatic patients. However, a variety of novel parameters also brought uncertainty regarding the clinical relevance of these indices, as well as the necessity for their validation in everyday practice. The aim of this review is to summarize the prevalence of HF in patients with severe AS and elaborate on the diagnostic challenges and advantages of comprehensive multimodality cardiac imaging to identify the patients that may benefit from surgical or transcatheter aortic valve replacement, as well as parameters that may help during follow-up.

## 1. Introduction

Aortic stenosis (AS) together with mitral regurgitation represents the most frequent valvular heart disease in the Western world [1]. In the natural history of AS, left ventricular hypertrophy (LVH) certainly remains the main compensatory mechanism that provides reduction of wall stress [2]. However, LVH eventually induces elevation of LV filling pressure that is retrogradely transferred to pulmonary circulation, causing dyspnea and ultimately cardiac pulmonary oedema in AS patients, even in conditions of preserved LV ejection fraction (LVEF) [2]. These patients are obviously developing an entity known as heart failure with preserved ejection fraction (HFpEF). Nevertheless, natural progression of AS includes progressive cardiomyocyte death and consequent fibrosis that replaces myocardial muscle and leads to the reduction of LVEF and development of heart failure with reduced ejection fraction (HFrEF) [2]. AS is often accompanied by coronary artery disease, arterial hypertension, diabetes, kidney disease, and other valvular diseases that can also contribute to the development of HFpEF or HFrEF [3].

The diagnosis of AS severity in HF patients nowadays is particularly challenging because of new entities such as low-flow low-gradient and ‘paradoxical’ low-flow low-gradient AS, which completely changed our perspective about AS patients and particularly about the therapeutic approach in these patients [4,5]. The prevalence of patients with severe AS and HF significantly varies between different studies and registries (10–35%) [6]. In the last couple of years, new data about severe AS in patients with different levels of LVEF or only in patients with HF have appeared and shed new light on the prognosis and treatment of these patients [7,8,9].

The importance of imaging and hemodynamic parameters in prognosis is even more intriguing because they can provide risk stratification of AS patients prior to the intervention (surgical or catheter) and indicate which patients need close follow-up. The best approach that will provide the most accurate information is certainly multimodality imaging. The updated classification of AS takes into account hemodynamic parameters instead of simple AV area and gradients. However, it is still unknown whether additional parameters, besides known echocardiographic- and CT-derived indices, should be used for more detailed evaluation of AS patients and particularly those with moderate and moderate-to-severe AS. Additionally, the long-term prognosis in patients with these new entities, particularly with low-flow low-gradient and paradoxical low-flow low-gradient AS, has not been fully understood so far.

This clinical review will summarize the current evidence about the multimodality imaging techniques and parameters that are used in diagnosis and prognosis of patients with severe AS and HF (HFpEF and HFrEF), as well as the strengths and weaknesses of these methods in everyday clinical circumstances.

## 2. Prevalence of Heart Failure in Patients with Severe AS

Heart failure in AS patients has long been defined by LVEF < 40% and more recently by LVEF < 50%. Therefore, the largest amount of available data is focused of HFrEF. Henkel et al. found symptomatic LV dysfunction (LVEF < 50%) in 24% patients with severe AS, whereas the prevalence of asymptomatic LV dysfunction was 0.4% [10]. The Scottish study involved 13,220 AS patients, of whom 25.1% had HF [11], while the Placement of Aortic Transcatheter Valve (PARTNER) trial reported 23% of patients with severe AS and LVEF < 50% [12]. In the large German aortic valve registry that includes severe AS patients who underwent surgical replacement or transcatheter aortic valve replacement (TAVR), LVEF < 50% was found in 15.8% of patients who underwent surgery and in 13.9% of patients who underwent TAVR [13]. A recent study that involved 708 patients with the whole range of LVEF found LVEF < 40% in 4.8% and 40% < LVEF < 50% in 11.7% patients with severe AS who underwent TAVR [7]. An older investigation performed in the pre-TAVR era that included 453 medically treated patients with severe AS showed that 35% of patients had LVEF < 40%, 24% patients had LVEF < 30% and 12% patients had LVEF < 20% [14]. Nowadays, there are studies that have investigated only HF patients (HFrEF, HFmrEF and HFpEF) with severe AS and the outcome after TAVR [9,15].

## 3. Heart Failure and Severe Aortic Stenosis—Difficulties in Diagnosing

Transvalvular mean gradient and aortic valve area (AVA) have been used as the main echocardiographic parameters for determination of AS severity for a long time. However, in patients with reduced LVEF, there is as discrepancy between relatively low gradients and significantly reduced AVA, which is why cardiac output assessed by indexed stroke volume was included in the assessment of AS severity. The poor outcome of patients with low-flow low-gradient AS, defined as AVA < 1 cm^2^, mean transvalvular aortic gradient < 40 mmHg, Svi < 35 mL/m^2^ and LVEF < 50%, emphasized the importance of detection of these patients [16]. More recent studies showed that patients with “paradoxical” low-flow low gradient AS (AVA < 1 cm^2^, mean transvalvular aortic gradient < 40 mmHg, Svi < 35 mL/m^2^ and LVEF > 50%) have the same unfavorable outcome [17]. The latest classification involved patients with normal-flow low-gradient severe AS (AVA < 1 cm^2^, mean transvalvular aortic gradient < 40 mmHg, Svi > 35 mL/m^2^ and LVEF > 50%), which seems to represent the most prevalent form of low-gradient AS [18]. The therapeutic approach is clear in patients with severe AS with high gradients as well as in low-flow low-gradient situations with preserved LVEF; however, in patients with preserved LVEF and low gradients (low- or normal-flow), it still remains controversial [19].

The appropriate diagnosis of low-gradient AS is crucial, and the low-dose dobutamine stress echocardiographic test is of the greatest importance in patients with low-flow low-gradient situations and preserved LVEF because it provides elevation of cardiac output by improvement of myocardial contractility and increased stroke volume. This is the best method to differentiate between true-severe and pseudo-severe AS [1,20]. LV flow reserve is preserved if stroke volume increases ≥20%. In true severe AS, mean transvalvular gradient increases (>40 mmHg), as well as stroke volume, whereas AVA remains reduced (≤1.0 cm^2^). In pseudo-severe AS, the improvement in LV stroke volume induces an increase in AVA (>1.0 cm^2^) and an insignificant increase, if any, in the mean transvalvular gradient (<40 mmHg) [1]. In paradoxical low-flow low-gradient AS, dobutamine stress echocardiography might add significant information on the differentiation between true and pseudo-severe stenosis [21]. However, one needs to take into consideration that there is a risk of hemodynamic deterioration in these patients while undergoing stress echocardiography due to an often-present concentric hypertrophy of the left ventricle. Nevertheless, some data showed no association between AS grading using dobutamine test and outcome, regardless of type of AS treatment (surgery vs. TAVR) [22]. Parameters obtained by dobutamine stress test (AVA and mean gradient) are flow-dependent and therefore, could not correctly classify AS severity and predict outcome [23]. This study actually demonstrated that projected AVA, a flow-standardized effective orifice area (EOA), provides incremental and superior diagnostic and prognostic information [23]. These findings imply that parameters obtained by dobutamine stress echocardiographic test and criteria proposed in the guidelines may lack sensitivity and possibly underestimate the actual AS severity.

In patients with low-gradient AS, other imaging modalities could significantly help. Computed tomography (CT) provides accurate evaluation of aortic valve calcification burden, which is associated with high sensitivity and specificity to identify patients in this group with true severe AS [24,25]. This is the reason why aortic valve calcium score is used for differentiation between true-severe and pseudo-severe AS, which is essential in HF patients. The lower threshold for aortic valve calcium score used for distinction between two AS entities is ≥1200 AU in women and ≥2000 AU in men [26]. Nowadays, CT is also used for reclassification of AS severity by fusion with echocardiography, which is particularly important in low-gradient AS patients [27]. Namely, the largest problem in the echocardiographic calculation of AVA is the premise that the left ventricular outflow tract (LVOT) is circular and not elliptic in the majority of patients. Therefore, CT or 3D echocardiography might be used for accurate anatomical evaluation of the LVOT area that would be incorporated into the existing continuity equation and possibly provide more accurate AVA than echocardiography. The fusion of CT and transthoracic echocardiography to calculate indexed AVA reclassified one-third of patients with low-gradient AS to moderate AS [27]. Nevertheless, this reclassification did not change the clinical outcome, even though patients reclassified to moderate AS with a LVEF <50% had worse outcomes owing to excess non-cardiac mortality [27]. This has particular clinical importance in HF patients with low-gradient AS, where AS can often be overestimated.

Cardiac magnetic resonance (CMR) offers the possibility to study markers of myocardial impairment that can impact the outcome of AS patients. Late gadolinium enhancement accurately identifies regional fibrotic tissue (scarring), which is frequently seen in patients with moderate and severe AS, but not diffuse interstitial fibrosis, which is typical for earlier stages of AS. Therefore, it might be helpful in the prediction of outcomes in patients with moderate and severe AS [28]. However, CMR-derived T1 mapping has enabled the quantification of diffuse interstitial fibrosis and might help also at earlier stages of disease. HF patients develop interstitial fibrosis, irrespective of AS, and therefore, it is not possible to distinguish tissue changes caused by HF from those induced by AS. Nevertheless, both conditions contribute to excessive fibrosis and further deterioration of LV function. Additionally, the existence of this level of interstitial myocardial fibrosis might help in reaching decisions to intervene even in patients with moderate-to-severe AS with AV area between 1.0 and 1.1 cm^2^.

## 4. The Role of Imaging in Prediction of Outcome in AS Patients with HF

Cardiac imaging in the assessment of AS severity and prediction of outcome long relied only on echocardiographic parameters. They included indices of LV structural and functional remodeling (LV mass index, LVEF, parameters of LV diastolic function). Development of echocardiographic techniques involved other indices, primarily the set of parameters of cardiac mechanics, starting with global longitudinal strain.

CT can also provide information about LV structure and function, but much more important are data on the aortic valve calcium burden, which showed significant predictive value in AS patients. CMR is useful for LV structural and functional information, but it is much more important for the evaluation of tissue characterization that cannot be provided with other imaging techniques. Nuclear medicine (PET/CT and PET/MRI) might be useful in the assessment of vascular inflammation in AS patients, but its role is still to be determined. Table 1 provides a practical summary of all technical and clinical advantages and limitations of each imaging method.

## 5. Echocardiography

A meta-analysis that included 7673 patients with severe AS undergoing TAVR showed that patients with LVEF < 30% had 60% higher 1-year mortality than those with LVEF > 50%, and even patients with LVEF < 50% had 52% higher mortality than patients with LVEF > 50% (HR 1.60; 95% CI: 1.19–2.16 and HR 1.52; 95% CI: 1.31–1.76, respectively) [29]. Low-gradient (<40 mmHg) AS was also related to a 60% higher mortality than high-gradient (>40 mmHg) AS 1 year after TAVR (HR 1.60; 95% CI: 1.30–1.97). Similar results were obtained for reduced stroke volume in comparison to normal stroke volume (HR 1.59; 95% CI: 1.25–2.05) [28]. Another meta-analysis including 14,099 patients after TAVR showed that reduced LVEF was not related to the 30-day mortality, but was related to the mid-term mortality in patients undergoing TAVR [30]. Patients with low-flow low-gradient AS had a higher 30-day and mid-term mortality compared to other AS patients. Subgroup analysis stratified AS patients by LVEF and reported consistent results in patients with LVEF < 30%, LVEF < 50%, and LVEF < 40% [29]. The authors also found that LVEF increased 8–10% after TAVR, which represents a remarkable improvement.

Investigations showed the association between LV global longitudinal strain (GLS) and cardiovascular events in asymptomatic patients with moderate and severe AS, which was independent of other echocardiographic and clinical parameters [31]. Vollema et al. reported that LV GLS significantly decreased over 12-month follow-up while LVEF remained unchained in asymptomatic patients with severe AS [32]. Patients with impaired LV GLS at baseline (>−18.2%) had a higher risk for developing symptoms and necessity for aortic valve replacement during follow-up in comparison with patients with higher values of LV GLS [32]. The study that compared LV changes after TAVR in patients with different LVEF found significant improvement in LVEF and GLS in patients with LVEF < 40%, whereas there was no change in patients with baseline LVEF of 40–55% [33]. Interestingly, the authors found deterioration of both parameters in patients with LEF > 55%. Kamperidis et al. included only patients with low-flow low-gradient severe AS irrespective of LVEF and reported significant improvement of LV GLS in both groups, even though GLS was significantly lower in patients with reduced LVEF (<50%) [34]. Baseline GLS but not LVEF was independently associated with GLS improvement 12 months after TAVR.

**Figure 1:**
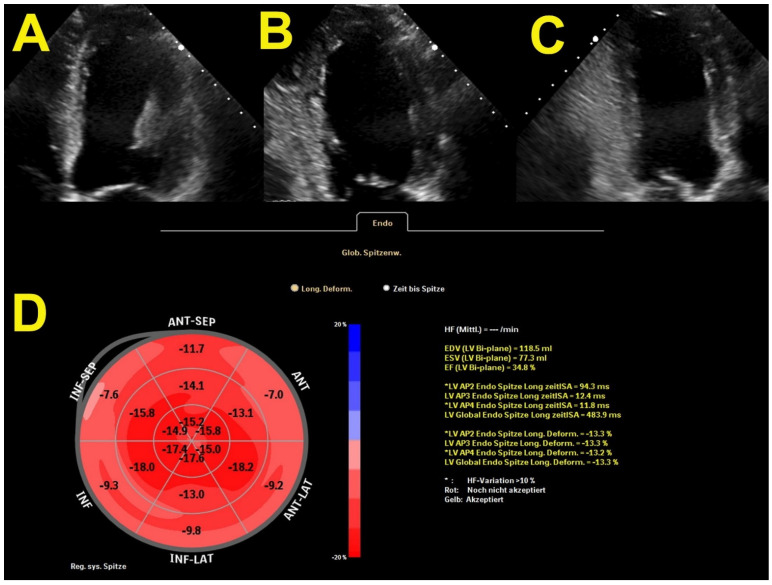
Echocardiographic assessment of left ventricular longitudinal strain with bullseye presentation. (**A**) 4-chamber view, (**B**) 2-chamber view, (**C**) 3-chamber view, (**D**) bullseye presentation of longitudinal strain of each myocardial segment. EDV—endi-diastolic volume, ESV—end-systolic volume, EF—ejection fraction, LV—left ventricicle, AP2—two-hamber view, AP3—three-chamber view, AP4—four-chamber view, ANT-anterior, SEP-septum, LAT-lateral, INF-inferior.

GLS has been proven to be a better predictor of myocardial recovery and symptomatic improvement than LVEF after TAVR in patients with severe AS with preserved and reduced LVEF [35]. A recent study showed that baseline twist and torsion add incremental prognostic value to GLS and LVEF in patients with severe AS who underwent TAVR [36]. Figure 1 illustrates a case of reduced GLS in patients with concomitant AS and HF.

The main advantages of echocardiographic assessment are its extensive availability and well-known parameters that should be measured and followed, as well as a significantly higher number of clinicians with the necessary expertise to perform this examination and a large body of data that established echocardiography as the gold standard in AS evaluation, including HF patients. However, there are several important limitations that have not yet been overcome with the echocardiographic approach. This refers mainly to the poor acoustic windows in a considerable number of patients, geometric assumptions regarding the shape of LVOT and often artifacts due to severe AV calcification.

## 6. Cardiac Magnetic Resonance

In a small study that used CMR for the assessment of LV remodeling in patients with severe AS who were treated surgically and interventional, the authors revealed that both groups were similar with respect to baseline and early post-procedure LVEF [37]. However, baseline LV GLS was significantly lower in TAVR patients compared to that in surgical patients. Early post-procedure, LV GLS significantly improved in TAVR patients, whereas it significantly worsened in surgical patients. After a long follow-up, both TAVR and surgical groups showed a significant reduction of LV mass and a significant improvement of LV myocardial multidirectional strain (longitudinal, circumferential and radial) [37].

**Figure 2:**
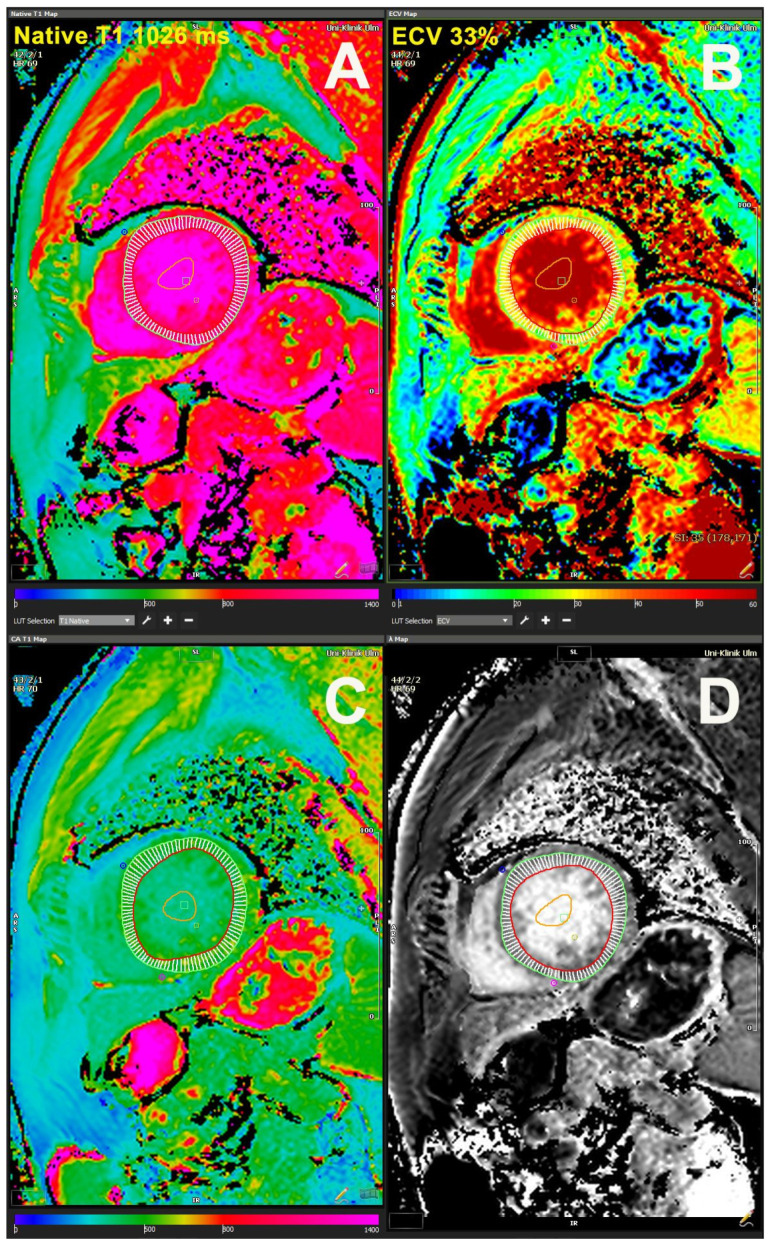
T1 mapping and extracellular volume fraction obtained by cardiac magnetic resonance. (**A**) native T1 mapping obtained before contrast, (**B**) extracellular volume fraction, (**C**) post-contrast T1 mapping obtained after contrast injection, (**D**) partition coefficient (λ) map, which is often used as a surrogate for ECV (extracellular volume fraction).

Tissue characterization obtained by CMR provides significant information about LV remodeling in AS patients. Lee et al. showed that a high native T1 value together with the presence and extent of LGE was an independent predictor of poor prognosis in patients with severe AS and preserved LVEF, whereas LVEF did not show important predictive value [28]. The T1 value increases with AS severity, together with morphological and functional LV alterations [38]. Myocardial fibrosis measured by T1 time correlated significantly with LV remodeling parameters (indexed LV mass and diastolic volume) and LV systolic function indexes (LVEF and GLS). Interestingly, myocardial fibrosis was higher in low-flow low-gradient AS than in the other types of AS, whereas the level of diffuse myocardial fibrosis was higher in patients with “paradoxical” low-flow low-gradient and high-gradient AS [38]. Figure 2 provides T1 mapping in a patient with AS and HFpEF and shows increased values of ECV in this patient.

A large study that included 440 patients with severe AS showed that myocardial fibrosis measured by extracellular volume fraction (ECV) correlated with markers of LV decompensation, LV mass, left atrial volume, New York Heart Association functional class III/IV, LGE, and lower LVEF [39]. During the 3.8-year follow-up, ECV was associated with cardiovascular and all-cause mortality. A recent study revealed significant reduction of myocardial fibrosis and improvement in LV systolic function in patients with severe AS after surgical replacement of the aortic valve [40]. The patients with improved T1 times after AV replacement had significantly higher improvement of LVEF and better outcome than those with extensive myocardial fibrosis after surgery [40].

The main advantages of CMR are the high-quality image, more accurate evaluation of LV function and possibility for tissue characterization. The limited possibility of accurate hemodynamic assessment (flow and gradients), deficient access, high-level training requirements for personnel, high costs, relatively long examination, problems with claustrophobia and incompatibility with metal implants represent important disadvantages of this diagnostic approach.

## 7. Computed Tomography

CT has been used for a long time for anatomical assessment and planning of TAVR. However, some parameters such as calcium burden of the aortic valve and more recently CT-derived GLS and CT-derived extracellular volume demonstrated significant prognostic value particularly in patients with low-flow AS [24,25,41,42]. Recent study identified a new parameter—the time to peak contrast enhancement from the test bolus images that correlates very well with bi-ventricular function and may accurately predict reduction in LV and RV function [43]. The authors even showed gradual changes in this parameter from patients with severe AS and LVEF > 50%, across those with 40% < LVEF < 50%, to those with LVEF < 40% [43].

**Figure 3:**
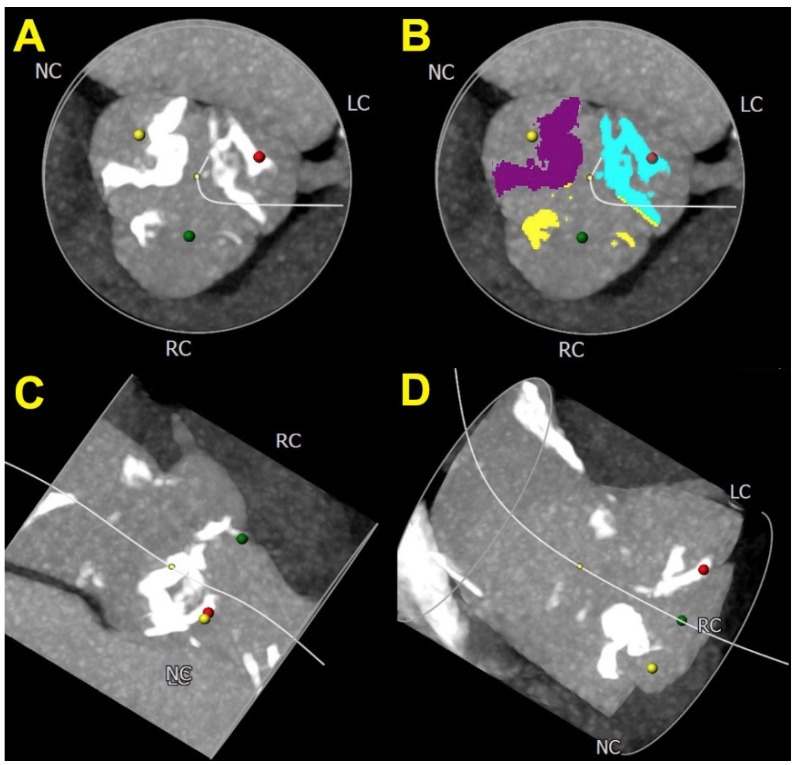
Computed tomography preparation for TAVI depicting very calcified aortic valve in different projections. LC—left coronary cusp, NC—non-coronary cusp, RC—right coronary cusp, (**A**) calcifications on each cusp of the aortic valve, (**B**) calcification depicted in different colors to facilitate the calculation of aortic valve calcification score, (**C**) aortic valve at the particular position provides better visualization for TAVI (RAO 99°, caudal 51°), (**D**) aortic valve at the particular position to provide better visualization for TAVI (RAO 7°, caudal 1°).

**Figure 4:**
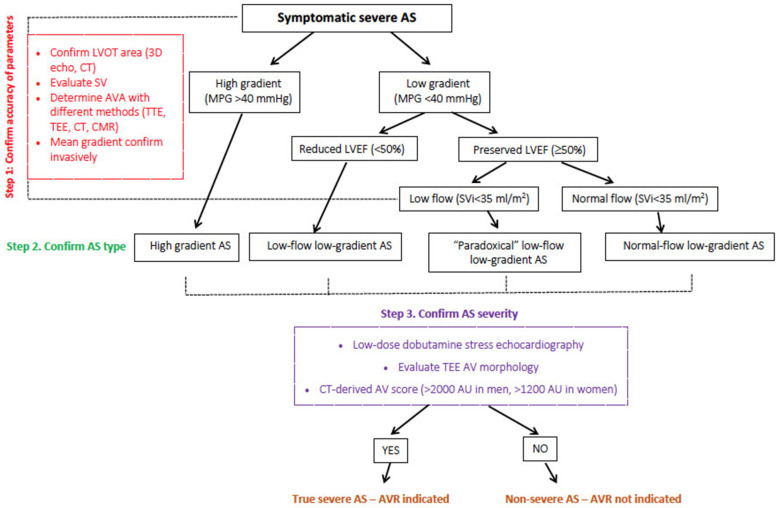
Flowchart for the diagnostic management of AS in patients with heart failure. AS—aortic stenosis, AVA—aortic valve area, LVEF—left ventricular ejection fraction, LVOT—left ventricular outflow tract, MPG—mean pressure gradient, SVi—stroke volume index, TEE—transesophageal echocardiography, CT—computed tomography, CMR—cardiac magnetic resonance, TTE—transthoracic echocardiography, AVR—aortic valve replacement, AU—unit used in CT.

The aortic valve calcium score was the first CT-derived prognostic parameter in symptomatic and asymptomatic patients with AS, and it has been used in decision-making, particularly in the latter group. It is sex-specific and predicts the severity of AS with high accuracy [44]. The major importance of the AV calcium score in the prediction of long-term mortality was reported in patients with low-flow low-gradient AS whose average LVEF was 21 ± 4.6% [45]. However, recent studies raised the question about the efficacy of the AV calcium score to predict major cardiovascular events in patients with the newer generation of TAVR devices [46]. Figure 3 presents an assessment of the AV calcium score and CT preparation for TAVR, and Figure 4 represents a proposed diagnostic flowchart that might be used in the clinical assessment of patients with AS and HF.

Reclassification of AS severity using a fusion approach that includes CT and echocardiographic data and hybrid continuity equation placed 30% of severe cases into the group of moderate AS, and the most affected were low-gradient AS patients (37% low-flow low-gradient and 59% normal-flow low-gradient AS) [47]. However, there was no difference in 2-year mortality risk between reclassified moderate and severe AS patients after TAVR [47].

Fukui et al. showed that reduced LV GLS in patients with severe AS and normal LVEF who underwent TAVR had higher mortality and worse composite outcome (mortality and hospitalization due to HF) than those with preserved GLS [41]. Moreover, there was no difference in outcomes between patients with preserved LVEF and reduced GLS and patients with reduced LVEF, which underlines the predictive importance of the determination of LV mechanics in patients with severe AS [41].

A study that investigated low-flow low-gradient AS showed that CT-derived ECV was a good predictor of mortality and hospitalization due to HF in these patients over a period of 14 months irrespective of other clinical and echocardiographic parameters [42]. A similar investigation revealed that the ECV fraction correlated with functional status and markers of LV decompensation and predicted the 1-year composite adverse clinical outcomes [48]. Recently it was reported that CT-derived ECV is a predictor of mortality and hospitalization due to HF independently of LVEF, AV calcium score, subtype of AS, or other clinical and echocardiographic parameters [49].

The ability to assess AV calcification level, AV anatomy, and structure, as well as the possibility to evaluate coronary artery anatomy, are the main advantages of this imaging technique, while exposure to radiation and contrast agents with potential nephrotoxic effects and the necessity to maintain relatively stable cardiac rhythm during short recordings represent the main limitations of this technique.

## 8. Nuclear Medicine (PET/CT, PET/MRI and Scintigraphy)

Hybrid imaging solutions such as PET/CT and PET/MRI can now enjoy the benefits of both techniques—anatomical (CT and MRI) and molecular (PET) to provide a comprehensive imaging assessment of AS. ^18^F-FDG PET is a widely used tracer for visualization of vascular inflammation, and it has been shown that it is accumulated in the aortic valve in AS patients, which confirms a previous hypothesis that AS is an active inflammatory condition [50]. ^18^F-FDG uptake was associated with significant progression of AS severity over a follow-up period [50]. A follow-up study showed that both ^18^F-fluoride and 18F-FDG predicted AS progression and adverse outcomes [51]. However, the role of PET in AS is presently limited only to research.

PET/MR is a developing technique, combining the high temporal and spatial resolution of MRI with the sensitive molecular imaging of PET, which offers several important advantages over PET/CT—primarily tissue characterization, better motion correction and lower radiation exposure. This might be of great importance in patients with concomitant AS and amyloidosis who constitute a growing population of patients that frequently also have HF. However, of greater diagnostic and prognostic importance in these patients with overlapping AS and amyloidosis are bone and cardiac scintigraphy. PET and bone scintigraphy in the diagnosis of cardiac amyloidosis should follow comprehensive echocardiographic and CMR examinations, and clinicians should pay attention to some red flags in this diagnostic flow-chart, starting from non-cardiac amyloidosis clinical features, microvolted QRS complexes in ECG, LVH in echocardiography and CMR, and low values of GLS with a typical bullseye that shows apical sparing, to elevated T1 values and ECV in CMR.

Recent study showed that 1-year mortality was worse in all patients with transthyretin cardiac amyloidosis (ATTR) cardiomyopathy than in those with isolated AS (24.5% vs. 13.9%; *p* = 0.05) [52]. TAVR significantly improved survival in patients with AS and ATTR cardiomyopathy, and survival of ATTR patients after TAVR did not differ from patients with isolated AS. Even though the prevalence of cardiac amyloidosis among patients with severe AS is high, it does not affect mortality after TAVR, but it is associated with significantly higher rates of hospitalization due to HF [53]. This is understandable if we keep in mind that the majority of patients with cardiac amyloidosis have at least HFpEF, if not HFrEF. Castano et al. reported that ATTR was diagnosed in 16% of patients with severe AS undergoing TAVR and was particularly associated with low-flow low-gradient AS with mildly reduced LVEF [54].

This is the least investigated imaging technique in evaluation of AS in HF patients, and therefore, only limited data are available. The main advantages are the assessment of AV inflammation and accurate LVEF. On the other hand, radiation exposure, high costs, and low availability are the important disadvantages of this imaging approach.

## 9. Conclusions

The assessment of AS severity in patients with HF is challenging, but nowadays the multimodality cardiac imaging approach provides a comprehensive evaluation of all anatomical and functional features not only of the aortic valve, but also of the myocardium, which is significantly impaired in patients with HF. Echocardiography remains the gold standard imaging tool for diagnosis and follow-up of AS patients. Nevertheless, CT and CMR should be used in everyday clinical practice and clinical decision-making, not only because of the accurate morphological data that can be obtained and that are necessary for the planning of TAVR, but also due to the valuable information about myocardial fibrosis and tissue characterization that have proved to have significant predictive value in AS patients who undergo TAVR or surgical replacement. This is particularly important in the challenging cases with concomitant AS and HF or moderate-to-severe AS where CT and CMR could significantly help clinicians to refer their patients for AV replacement. In the last few years, cardiac amyloidosis has received particularly high attention in patients with severe AS, and therefore, CMR and scintigraphy should also be involved, when necessary, in the diagnostic flow-chart of patients with AS. Despite the many imaging techniques and parameters that are used for the evaluation of severity or prognosis in AS patients, there are still many uncertainties regarding the appropriate set of parameters that should be clinically used in specific subsets of AS patients with HF.

## Figures and Tables

**Table 1:** The strengths and limitation in assessment of aortic stenosis and left ventricular structure and function using different imaging modalities.

	2DE (TTE and TEE)	3DE (TEE)	CMR	CT	FDG-PET
Availability	High	Moderate-High	Moderate	Moderate-high	Low
Cost	Low	Low	High	Moderate	High
Typical scan duration (min)	20–35	5–10 *	40–60	10	120
Preparation for test	No	No	No **	No	Yes
Safety	High	High	Contrast in patients with renal failure Contraindicated in patients with metallic implants Restricted to hemodynamically stable patients	Radiation Contrast in patients with advanced renal failure	Radiation Allergic reactions
Problem with imaging window	Present	Present	Absent (if no implants)	Absent	Absent
Temporal resolution	++++	+++	+++	++	+
Spatial resolution	+++	++	+++	++++	++
Contrast to noise ratio	+++	++	++++	+++	++
Authentic 3D imaging	No	Yes	Only in selected sequences	Yes	Yes
Possibility for real-time 3D imaging	No	Yes	No	No	No
Assessment of AV structure	++	+++	++	++++	+
Assessment of AV calcification	++	++	+	++++	+
Assessment of AV inflammation	+	++	+	+	++++
Assessment of LV tissue characterization	-	-	++++	++	+
Anatomic assessment of AVA	++	+++	++	+++	Not used
Assessment of low-gradient AS	++++	++	++	+++	Not used
Evaluation of LV function	++	+++	++++	++	+++
Evaluation of LV strain	++++	+++	+++	++	-
Major limitations	Geometric assumptions of LVOT shape Poor acoustic windows Artifacts due to AV calcification	Stable cardiac rhythm Artifacts due to AV calcification	Stable cardiac rhythm Cost Low availability Patients with metallic implants Claustrophobia	Ionizing radiation Potentially nephrotoxic contrast Stable cardiac rhythm with	Cost Low availability Ionizing radiation Claustrophobia

+—low, ++—moderate, +++—high, ++++—very high, *—additional time necessary after 2DE, **—preparation for CMR is not necessary if patient does not undergo stress CMR test, 2DE—two-dimensional echocardiography, 3DE—three-dimensional echocardiography, AV—aortic valve, AVA—aortic valve area, CMR—cardiac magnetic resonance, CT—computed tomography, FDG-PET—fluorodeoxyglucose (FDG)-positron emission tomography, LV—left ventricle, LVOT—left ventricular outflow tract.

## Data Availability

Not applicable.
